# Bilateral undisplaced insufficiency neck of femur fractures associated with short-term steroid use: a case report

**DOI:** 10.1186/1752-1947-2-79

**Published:** 2008-03-11

**Authors:** Sabhat Gurdezi, Ravi K Trehan, Mark Rickman

**Affiliations:** 1 Registrar, Department of Trauma and Orthopaedics, St Georges Hospital, Blackshaw Rd, London, SW17 0QT, UK; 2 Study was carried out at St George's Hospital, Tooting, London, UK

## Abstract

**Introduction:**

We present an interesting and unusual case of a 61-year-old woman with bilateral, undisplaced, stress neck of femur fractures associated with short-term steroid use. Insufficiency fractures of the neck of femur without preceding trauma have been described in the literature, although bilateral involvement is infrequent. These fractures have been associated with strenuous exercise, seizures, renal osteodystrophy, fluoride treatment, long-term corticosteroid use, amenorrhoea, abnormal anatomy and osteomalacia due to nutritional and/or hormonal factors.

**Case Presentation:**

The case we present differs from other published reports, in that the patient's symptoms developed acutely after only a short course of steroids and with no associated trauma or strenuous exercise. It is also the only case described where no operative intervention was required.

**Conclusion:**

Our case reiterates the importance of considering insufficiency or stress fractures in high-risk patients who present with musculoskeletal pain. Institution of bone protection should also be considered in these patients. Morbidity related to delayed treatment has been well documented, so a high level of clinical suspicion is imperative.

## Introduction

We present an interesting and rare case of a woman with bilateral, undisplaced, stress neck of femur fractures associated with short-term steroid use. Insufficiency fractures of the neck of femur without preceding trauma have been described in the literature, although bilateral involvement is infrequent. The femoral neck is the most common site of fatigue and insufficiency fractures in the femur [1]. These fractures have been associated with strenuous exercise, seizures, renal osteodystrophy, fluoride treatment, long-term corticosteroid use, amenorrhoea, abnormal anatomy and osteomalacia due to nutritional and/or hormonal factors. The case we present differs from other published reports, in that the patient's symptoms developed acutely after only a short course of steroids and with no associated trauma or strenuous exercise. It is also the only case described where no operative intervention was required.

## Case Presentation

**Figure 1:**
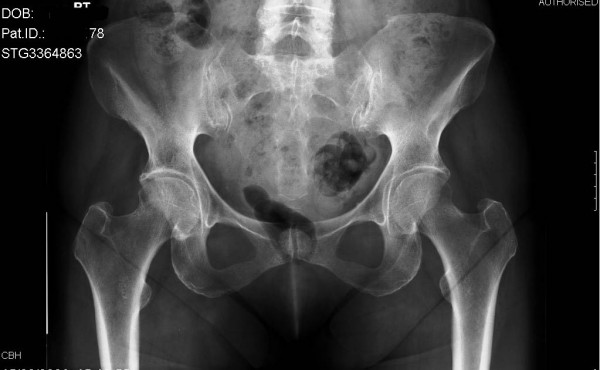
Plain radiograph taken at the time of initial presentation and reported as 'no bony injury'.

**Figure 2:**
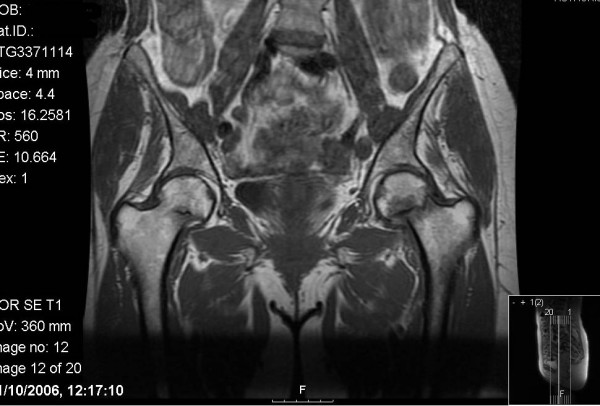
**T1 weighted MRI showing bilateral undisplaced neck of femur fractures.** This investigation was performed six weeks after the onset of symptoms.

**Figure 3:**
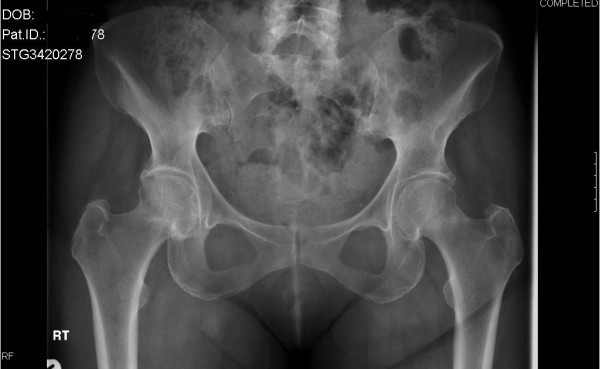
Three month follow-up plain radiograph showing healed undisplaced neck of femur fractures.

A 61-year-old woman presented to our hospital after waking with pain in her left hip. She had been taking oral prednisolone (30 mg twice daily) for the previous three weeks for recently diagnosed acute nephritis. Plain radiographs at this time showed no bony injury (Figure 1). Within one week the pain became bilateral and this previously independently mobile patient required assistance with two sticks. As symptoms did not improve further imaging was arranged. An MRI showed bilateral, subcapital, undisplaced fractures of both femoral necks (Figure 2). On review by the orthopaedic team, the patient's symptoms had improved and clinical examination of the hip showed only slight discomfort on internal rotation. As this was now six weeks since the onset of symptoms and the fractures had remained undisplaced despite mobilising, conservative management with surveillance was deemed appropriate. It was decided that if there was any displacement or signs of non-union, further treatment would be offered. At reviews at 3, 6 and 12 months, radiographs showed healed undisplaced bilateral neck of femur fractures (Figure 3). The patient had a full range of movement in both hips and was mobilising normally without aids, and therefore declared clinically healed, at three months.

## Discussion

Corticosteroids induce osteopenia by both direct and indirect methods. Direct effects include: suppression of osteoblastic activity, reduction of intestinal calcium absorption, increased urinary calcium excretion and decreased renal tubular calcium absorption. This in turn leads to secondary hyperparathyroidism causing increased bone resorption and turnover [2].

Insufficiency fractures occur in patients with intrinsic or iatrogenic osteopenia under normal loading conditions. These fractures have been described in patients on long term corticosteroid treatment, those who have renal osteodystrophy, patients who have received fluoride treatment, amenorrheic athletes and those who have undergone pelvic irradiation [3]. Fatigue fractures on the other hand occur as a result of excessive and usually repetitive strain on normal bones. These fractures are common in endurance athletes, military personnel and people with epilepsy. Fatigue fractures can also result from normal stress forces through abnormal anatomy. Femoral neck fractures have been described post bilateral total knee replacement due to the resultant alteration in normal leg axis [4], and also due to bilateral protrusio acetabuli in a patient with Marfan's syndrome [5].

Previous studies looking at corticosteroid use and neck of femur insufficiency fractures involved patients on long term courses of treatment. Zuckerman et al [6] described bilateral femoral neck stress fractures in a woman who had received a three month course of steroids five years prior to the onset of symptoms. Austin et al [7] described a 67-year-old patient who had been on steroids for several years. And Haddad et al [8] described a woman who had been on high dose corticosteroids for at least two months. In all of these cases operative intervention was required or recommended. The patient in our case report differs in that she presented with symptoms after only three weeks of steroid treatment and her fractures remained undisplaced despite mobilisation, and so did not require surgical fixation.

Fortunately, our patient did not come to any harm from the delay in diagnosis, but morbidity with delayed treatment has been well documented, so a high level of clinical suspicion is imperative. Many studies have looked at the sensitivity of various imaging modalities for these fractures [1,9], with MRI being the most specific and bone scans the most sensitive. Investigation of bone mineral density and initiation of bone protection should always be considered in people in high risk groups.

## Conclusion

This case report reiterates the importance of considering stress fractures in high risk patients who present with musculoskeletal pain, even in those that may have only been on a short course of steroids. Plain radiographs may falsely reassure the clinician; therefore further investigation with other imaging modalities is necessary to avoid delayed diagnosis and any ensuing morbidity.

## Competing interests

The author(s) declare that they have no competing interests.

## Authors' contributions

SG carried out the initial literature review, collation of data, drafting of the original manuscript, revision of the manuscript and submission of the final manuscript. RT was involved in the conception of the case report, the literature review, revision of manuscript and approval of the final manuscript. MR was the clinical lead for this case report and clinical assessor of the patient, assisting with conception of the case report and approval the final manuscript.

## Consent

Written informed consent was obtained from the patient for publication of this case report and all accompanying images. A copy of the written consent is available for review by the Editor-in-chief of this journal.
